# Coping style and social support as modifiable mediators between personality and self-perceived speech handicap in head and neck cancer patients with tracheostomy: a cross-sectional chain-mediation study

**DOI:** 10.3389/fpsyg.2026.1920840

**Published:** 2026-09-08

**Authors:** Yinfeng Li, Jie He, Yan Zhang, Qinghua Jiang, Yin-Ping Zhang

**Affiliations:** 1 Faculty of Nursing, Xi’an Jiaotong University Health Science Center, Xi'an, China; 2 Department of Head and Neck, Sichuan Clinical Research Center for Cancer, Sichuan Cancer Hospital & Institute, Sichuan Cancer Center, University of Electronic Science and Technology of China, Chengdu, China

**Keywords:** head and neck neoplasms, personality, social support, speech handicap index, tracheostomy

## Abstract

**Objective:**

This study sought to elucidate the chain mediation effects of coping style and social support linking extraversion-introversion personality to self-perceived speech handicap in head and neck cancer patients with tracheostomy.

**Methods:**

A cross-sectional survey was conducted among 217 post-tracheostomy patients (January 2023–June 2024). Data collection was conducted through questionnaire surveys employing a general information questionnaire, the Eysenck Personality Questionnaire-Revised, Short Scale for Chinese, the Social Support Rating Scale, the Simplified Coping Style Questionnaire, and the Chinese version of the Speech Handicap Index. The outcome was the total SHI score, a patient-reported measure of self-perceived speech handicap; communication ability was not directly measured.

**Results:**

The mean score for extraversion-introversion personality traits among participants with tracheostomy was 51.798 ± 11.240, for positive coping style was 30.790 ± 7.240, for social support was 40.862 ± 9.024, and for the Speech Handicap Index was 73.230 ± 24.227. Speech handicap exhibited significant negative correlations (*p* < 0.01) with extraversion-introversion personality traits (*r* = −0.445), coping style (*r* = −0.410), and social support (*r* = −0.402). Both positive coping style and social support significantly mediated the association between extraversion-introversion personality traits and self-perceived speech handicap in head and neck cancer patients with tracheostomy, with mediation effect values of −0.682 and −0.425, respectively. Furthermore, a chain mediation effect was observed along the “positive coping style → social support” pathway (−0.201), accounting for 6.26% of the total effect.

**Conclusion:**

Path analysis identifies an associative relationship between extraversion-introversion personality traits and self-perceived speech handicap that is potentially explained by the chained mediating roles of coping style and social support. As patients’ extraversion-introversion personality traits are difficult to modify, optimization of coping style and enhancement of social support levels in clinical practice may constitute primary intervention strategies for reducing self-perceived speech handicap in these patients.

## 1 Introduction

Head and neck cancer constitutes the sixth most prevalent malignancy worldwide (Li et al., 2023). According to the most recent statistics from the National Cancer Center, approximately 144,600 new cases of head and neck squamous cell carcinoma were diagnosed in China in 2022, with nearly 80,500 deaths reported (Zheng et al., 2024). Patients with stage III and IV head and neck squamous cell carcinoma exhibit poor treatment outcomes, with a 5-year overall survival rate of only 40–50% (Cramer et al., 2019). In major head and neck cancer surgeries, the primary purpose of performing a temporary tracheostomy is to ensure a secure airway during the period of postoperative edema resolution (National Institute for Health and Care Excellence, 2015; Stafford et al., 2016). A multidisciplinary study investigating long-term benefits and risks indicated that approximately 69% of patients with head and neck cancer, including oral, hypopharyngeal, and laryngeal cancers, undergo tracheostomy during surgery (Marsh et al., 2019). Regardless of whether temporary or permanent, tracheostomy induces psychological vulnerability due to functional impairment and social isolation caused by the temporary loss of verbal communication (National Institute for Health and Care Excellence, 2015; Stafford et al., 2016). Communication difficulties, particularly impaired speech, are core challenges for head and neck cancer patients with tracheotomy, primarily attributed to disease characteristics (e.g., laryngeal/tracheal anatomical changes caused by tumor location, vocal cord damage from surgical resection) and tracheotomy-related airway modifications (Chauhan et al., 2022; Bray et al., 2020).

While disease and surgical factors are the primary determinants of speech function, accumulating evidence suggests that personality traits-especially Eysenck’s core dimensions (extraversion-introversion), may exert a significant regulatory or predictive effect on how patients perceive and cope with communication impairments (Swickert et al., 2002; Ioerger et al., 2024). The present study focuses on the speech component of these difficulties, operationalized as self-perceived speech handicap measured by the Speech Handicap Index. The head and neck organs determine functions such as breathing, speech, and swallowing in patients. The occurrence of diseases, especially after tracheotomy, can affect patients’ speech function and social expression ability, leading to speech impairment and its psychosocial impact. In the context of early cancer screening and treatment, reducing psychosocial barriers such as fear of recurrence, social withdrawal, and speech difficulties, and improving quality of life, are recognized priorities in psycho-oncology (National Institute for Health and Care Excellence, 2015; Stafford et al., 2016; Marsh et al., 2019; Yuan et al., 2020).

Extraversion-introversion has been consistently linked to stressor appraisal, coping selection, and support mobilization (Connor-Smith and Flachsbart, 2007). Extraverted individuals tend to adopt approach-oriented coping and actively mobilize support networks, including adaptive communication strategies such as writing boards or speech-generating devices, whereas introverted individuals more often rely on avoidant or independent strategies and under-utilize available support (Connor-Smith and Flachsbart, 2007; Carver and Connor-Smith, 2010; Birkhaug et al., 2002).

Based on the theoretical considerations and empirical findings outlined above, we systematically reconstructed the logical relationships among core variables in head and neck cancer patients with tracheotomy. The foundation of the model is that disease and surgical characteristics (tumor location, surgical extent, tracheostomy status) are the primary objective determinants of speech impairment (National Institute for Health and Care Excellence, 2015). Second, personality traits play a regulatory role: they do not directly alter the anatomical and surgical causes of speech impairment, but they regulate patients’ adaptive responses to these difficulties. This is consistent with the framework of Eysenck’s Revised Personality Theory adopted in this study, which emphasizes that extraversion-introversion is a stable, heritable core trait that shapes individuals’ behavioral and emotional responses to environmental stressors (e.g., illness-related impairments) (Eysenck and Eysenck, 1985; Bolger, 1990). Speech impairment (whose psychosocial impact may be exacerbated or mitigated by personality traits) leads to reduced social interaction, which in turn increases social isolation and psychological vulnerability (Bolger, 1990). Previous research has further shown that following the onset of head and neck cancer and surgical intervention, patients’ personalities tend to gradually shift toward introversion, accompanied by heightened anxiety, diminished expressive ability, excessive sensitivity to external stimuli, and substantially reduced resilience (Bray et al., 2020). These negative states further affect patients’ coping strategies and, subsequently, their acquisition and utilization of social support (Chauhan et al., 2022; Birkhaug et al., 2002).

Existing studies have separately explored the relationships between personality traits and social support/coping strategies, or between self-perceived speech handicap and social isolation, but few have integrated these variables into a unified logical framework—especially ignoring the regulatory role of Eysenck personality traits in the “self-perceived speech handicap → social adaptation” process. This study analyzed the impact of extraversion-introversion personality traits on self-perceived speech handicap in this patient population, as well as the mediation effect of social support and coping style in the relationship between personality traits and self-perceived speech handicap, in order to inform perioperative nursing strategies that support postoperative communication.

This study was framed by Lazarus and Folkman’s transactional model of stress and coping, in which stable dispositions shape how individuals appraise a stressor, mobilize coping responses, and access social resources. Extraversion–introversion was treated as a relatively stable dispositional antecedent, and coping style and social support as potentially modifiable processes linking personality to self-perceived speech handicap (Lazarus and Folkman, 1984; Folkman and Moskowitz, 2004). We describe coping style and social support as modifiable because both can be altered by structured psychosocial nursing interventions, for example, coping-skills training, peer-support programs, and family-support mobilization, whereas personality traits are relatively stable. Modeling personality as a non-modifiable antecedent and coping style and social support as modifiable mediators serves two clinical purposes: it positions personality as a screening marker for identifying patients at elevated risk, and it quantifies how much of the personality–handicap association is transmissible through intervention-amenable pathways—information that bivariate association analyses cannot provide.

The specific mechanisms underlying each link of the chain, personality → coping style → social support → speech handicap, are elaborated below.

Mechanism 1: Personality on coping style. Extraverted patients appraise speech impairment more as a manageable challenge and engage in active, approach-oriented coping (e.g., learning alternative communication methods), whereas introverted patients more often adopt avoidant coping. We therefore hypothesized a positive association between extraversion and positive coping (H1).

Mechanism 2: Coping style on social support. Active, positive coping entails proactively seeking and utilizing support—sharing difficulties, accepting help, and using communication aids—thereby mobilizing the patient’s support network; avoidant coping reduces help-seeking and leaves support under-utilized. This is consistent with the “utilization of social support” dimension of the Social Support Rating Scale (SSRS). We hypothesized a positive correlation between coping style and social support (H2).

Mechanism 3: Social support on speech handicap. Consistent with the stress-buffering account of social support (Marsh et al., 2019), adequate social support may buffer the psychosocial impact of speech impairment—reducing social isolation, distress, and avoidance captured by the SHI—thereby lowering perceived handicap, whereas insufficient support may amplify these burdens. We therefore hypothesized a negative association between social support and self-perceived speech handicap (H3).

## 2 Study subjects and methods

### 2.1 Study subjects

A cross-sectional and correlational study was conducted and is reported in accordance with the STROBE statement for observational studies (Supplementary Checklist). Two hundred and twenty-eight head and neck squamous cell carcinoma patients were approached by convenience sampling between 30 January 2023 and 30 June 2024. Prior to the survey, the research objectives and significance were explained to the participants, and informed consent was obtained. Thyroid carcinoma is excluded from this statistic because it differs from other head and neck cancers in biology, prognosis, and surgical management, and rarely entails tracheostomy (Zheng et al., 2024). Inclusion criteria: (1) Diagnosed with head and neck cancer and underwent tracheostomy; (2) Age ≥ 18 years; (3) Absence of mental illness, psychological dysfunction, or cognitive impairment; (4) Voluntary participation in the study. Exclusion criteria: (1) Presence of concurrent malignant tumors; (2) Inability to cooperate due to other causes.

All eligible patients had undergone surgical treatment with tracheostomy. Patients managed with primary (chemo)radiotherapy were not included. This restriction was intentional, because surgery with tracheostomy constitutes a discrete clinical event shared by all participants, anchoring the onset of speech impairment and its assessment to a comparable recovery phase, whereas (chemo)radiotherapy produces speech impairment through different mechanisms (e.g., mucositis, edema, fibrosis) and along a gradual trajectory without a comparable onset point. Restricting the sample in this way enhanced internal homogeneity at the cost of external generalizability, which we address in the Limitations.

The sample size for chain mediation analysis was determined based on the requirements for detecting indirect effects. According to Fritz and MacKinnon (2007) (Fritz and MacKinnon, 2007), detecting a medium-sized indirect effect (*β* = 0.26) in a multiple mediation model with two sequential mediators requires a minimum sample size of approximately 200 to achieve 80% statistical power at *α* = 0.05. Given our final sample of 217, the chain mediation findings should be interpreted as exploratory. This sample size is nevertheless adequate for detecting the individual mediation pathways and the direct effect, which are the primary focus of this study. This study received approval from the SICHUAN CANCER HOSPITAL & INSTITUTE Ethics Committee (SCCHEC-02-2023-004). Each patient signed a paper-based informed consent form. This study was conducted as a single-center, cross-sectional, correlational survey without any intervention. As an observational study, trial registration was not required and was not performed.

### 2.2 Methods

#### 2.2.1 Research tools

##### 2.2.1.1 General information questionnaire

A self-designed questionnaire was employed, encompassing age, sex, educational level, marital status, monthly household income, method of payment for hospitalization expenses, current place of residence, employment status, and religious belief. These sociodemographic variables were collected because they are associated with social support availability, coping resources, and health literacy, and were therefore used as covariates in the mediation models to reduce confounding.

##### 2.2.1.2 Eysenck personality questionnaire-revised, short scale for Chinese (EPQ-RSC)

The EPQ-RSC was developed by Chinese psychologists Qian et al. (2000) on the basis of the Eysenck Personality Questionnaire-Short Scale (Eysenck and Eysenck, 1991). This instrument classifies human personality traits into four dimensions: Neuroticism (N), Extraversion (E), Psychoticism (P), and Lie (L). In this study, the Extraversion (E) scale was employed to assess patients’ extraversion–introversion personality traits. This scale consists of 12 items, among which 11 are scored positively (a response of “yes” is assigned 1 point, otherwise 0 points), while 1 item is reverse-scored (a response of “yes” is assigned 0 points, otherwise 1 point). Following summation of all item scores to obtain a total score, standardized T-scores were calculated according to age- and sex-based norms using the equation: T = 50 + 10 × (participant’s raw score − mean score of the participant’s group)/standard deviation of the group’s scores. Lower T-scores correspond to more introverted personality characteristics. Specifically, T < 38.5 indicates introverted personality; 38.5 ≤ T < 43.3 indicates a tendency toward introversion; 43.3 ≤ T ≤ 56.7 represents intermediate personality; 56.7 < T ≤ 61.5 indicates a tendency toward extraversion; and T > 61.5 indicates extraverted personality. In the current sample, the Cronbach’s α coefficient for the Extraversion subscale was 0.82.

##### 2.2.1.3 Social support rating scale (SSRS)

The SSRS was developed by Xiao (1994) and is primarily applied to evaluate individual social support status. The scale comprises 10 items organized into three dimensions: objective support, subjective support, and utilization of social support. Items 1–5 and 8–10 are rated on a 4-point Likert scale, whereas items 6–7 are scored according to the number of available sources, with zero points assigned when no sources are reported. The total score is obtained by summing all item scores, with higher totals reflecting greater levels of social support. In this study, the total social support score dimension was employed, and it exhibited a test–retest reliability coefficient of 0.920. The SSRS was selected because it is widely used and well-validated Chinese-language instrument for social support, with established reliability in Chinese oncology populations (Xiao, 1994).

##### 2.2.1.4 Simplified coping style questionnaire (SCSQ)

The SCSQ was developed by Yaning Jie (Xie, 1998) to evaluate coping tendencies that may arise when individuals encounter external stimuli. The SCSQ comprises a positive coping subscale (12 items, range 0–36) and a negative coping subscale (8 items, range 0–24). For the mediation analyses, the positive coping subscale score was used as the mediator variable. Both subscale scores are reported descriptively in Table 1. Only the positive coping subscale entered the mediation analyses, because the theoretical model concerns modifiable protective resources, and including both moderately correlated subscales would risk multicollinearity in a sequential model of modest sample size. Higher scores on the positive coping subscale reflect a stronger inclination toward positive coping (Cao et al., 2024; Song et al., 2016; Zhang et al., 2005). The Cronbach’s α coefficient for this variable was 0.905. The SCSQ was selected for its brevity, established reliability and validity in Chinese samples, and its wide use in Chinese oncology research (Xie, 1998; Cao et al., 2024; Song et al., 2016; Zhang et al., 2005).

**Table 1:** Correlations among extraversion-introversion, coping style, social support, and self-perceived speech handicap (*r*-values).

Variables	Extraversion-introversion	Coping style	Social support	Self-perceived speech handicap
Extraversion-introversion	1			
Coping style	0.481**	1		
Social support	0.386**	0.384**	1	
Self-perceived speech handicap	−0.445**	−0.410**	−0.402**	1
Mean	51.798	30.790	40.862	73.230
Standard deviation	11.240	7.240	9.024	24.227

**p < 0.01. SHI = Speech Handicap Index; higher SHI scores indicate greater self-perceived speech handicap. Following Cohen (1988), |*r*| ≈ 0.10, 0.30, 0.50 denote small, medium, and large effects; all observed correlations are in the medium range.

##### 2.2.1.5 Chinese version of speech handicap index (SHI)

The Chinese version of the SHI was employed for evaluation in this study. This scale was adapted and validated by Peixia Wu and colleagues in 2014 (Wu et al., 2014), with its reliability and validity examined. It remains the only comprehensive instrument documented in the literature that specifically captures the psychosocial impact of speech disorders in head and neck cancer patients. Speech problems after head and neck surgery comprise both organic (anatomical/surgical) and psychosocial components, and personality has documented associations with functional voice disorders. The SHI contains 30 items divided into two dimensions: speech severity and psychosocial impact. Each item is rated on a 5-point Likert scale ranging from “never” to “always,” corresponding to 0–4 points. In this study, communication was assessed only through the SHI, which captures speech-related handicap rather than the full range of verbal and non-verbal communication; accordingly, the findings pertain to self-perceived speech handicap. The total SHI score was applied, with higher scores indicating greater self-perceived speech handicap. In the current sample, the Cronbach’s α coefficient for the SHI total scale was 0.955.

It should be noted that the SHI is a patient-reported outcome measure rather than an objective assessment of speech function. According to the World Health Organization’s International Classification of Functioning, Disability and Health (ICF), impairment (e.g., reduced speech intelligibility resulting from anatomical and surgical changes) and the resulting activity limitations and participation restrictions (i.e., perceived handicap) are distinct levels of functioning: patients with comparable objective impairment may experience markedly different degrees of handicap, and this subjective component is precisely where psychosocial factors are expected to operate. Self-perceived speech handicap therefore constitutes a clinically meaningful endpoint in its own right (Wu et al., 2014; World Health Organization, 2001).

#### 2.2.2 Data collection methods

Questionnaires were administered through on site, with responses entered through the online Questionnaire Star platform. Patients were rigorously screened according to the inclusion and exclusion criteria, and questionnaires were distributed after informed consent had been obtained. All questionnaires were completed independently by the patients. For those unable to complete the forms themselves, a designated investigator read the items aloud and recorded the responses on their behalf. Each questionnaire was permitted to be completed only once. After collection, questionnaires were reviewed for missing items, and patients were guided to supplement incomplete information. Missing continuous data (≤10%) were imputed by mean substitution, which may underestimate variance and is acknowledged as a limitation. In total, 228 questionnaires were distributed, of which 217 were valid, resulting in an effective response rate of 95.2%.

Each questionnaire was administered on a single occasion during the patient’s postoperative hospitalization, while the tracheostomy tube was in place and before decannulation. The exact postoperative day of assessment was not recorded; however, restricting assessments to the in-hospital, cannulated period anchored all measurements to a comparable clinical phase of the postoperative course.

#### 2.2.3 Statistical methods

All original survey data from the study subjects were initially entered into Microsoft Excel to establish a structured database. Following database construction, data accuracy was verified using a “double-entry plus cross-validation” procedure, after which data cleaning was performed. Invalid samples with logical contradictions (e.g., scale scores exceeding theoretical ranges) were excluded to ensure that the final dataset satisfied statistical requirements.

After preprocessing, IBM SPSS 26.0 statistical software was employed to conduct descriptive analyses on data that met the analytical criteria. Categorical variables (e.g., patient sex and educational level) were summarized as “frequency (percentage, n/%)”; continuous variables (e.g., extraversion–introversion personality trait scores, social support scores, and SHI) were expressed as “mean ± standard deviation”.

Because all data in this study were obtained through questionnaire surveys, potential common method bias was considered. Therefore, Harman’s single-factor test was performed as an *a priori* control. Specifically, all scale items were subjected to unrotated exploratory factor analysis, and if the variance explained by the first common factor was <40%, the common method bias was regarded as weak, thereby supporting good data reliability. We also used the marker variable technique by including a theoretically unrelated variable (patient satisfaction with hospital meals) as a marker. The partial correlations among key variables remained significant after controlling for the marker variable, indicating that common method bias did not substantially inflate the observed relationships.

Continuous variables were tested for normality using the Shapiro–Wilk test, all approximated a normal distribution (all *p* > 0.05). Accordingly, Pearson product–moment correlation analysis was used to examine relationships among extraversion–introversion (independent variable), coping style and social support (mediators), and SHI score (outcome). This analysis clarified both the direction and strength of correlations, thereby establishing a foundation for subsequent mediation effect testing. Chained mediation analysis was performed using Model 6 of the PROCESS macro (version 4.1) for SPSS 26.0 (Hayes, 2017), adjusting for age, sex, marital status, educational level, residence, employment status, family income, with 5,000 bootstrap resamples and bias-corrected 95% confidence intervals; an indirect effect was considered significant when its CI excluded zero. Specific pathway tests included: ① the individual mediation effect of coping style between extraversion–introversion personality traits and self-perceived speech handicap; ② the individual mediation effect of social support between extraversion–introversion personality traits and self-perceived speech handicap; ③ the chain mediation effect of “extraversion–introversion personality traits → coping style → social support → self-perceived speech handicap.” Across all statistical analyses, *p* < 0.05 (two-tailed) was considered indicative of statistical significance (Fang et al., 2012).

## 3 Results

### 3.1 Common method bias analysis

As the data in this study were obtained through self-administered questionnaires, the results may be subject to common method bias. To assess this, the four scales were examined using Harman’s single-factor test. The variance explained by the first principal component was 31.962%, which falls below the threshold proposed by Zhou and Long (2004), indicating that common method bias can be regarded as weak when the variance explained by the first factor is under 40%. Therefore, it was inferred that the questionnaire survey results of this study are not markedly affected by common method bias.

The general demographic data of this study are displayed in Table 2. According to the Eysenck Personality Questionnaire Short Form E Scale, the distribution of results was as follows: 25 participants (11.52%) exhibited an introverted personality; 13 participants (5.99%) demonstrated a tendency toward introversion; 97 participants (44.7%) presented with an intermediate personality; 38 participants (17.51%) showed a tendency toward extraversion; and 44 participants (20.28%) exhibited an extraverted personality. Within this study, the intermediate personality type represented the largest proportion, followed by the extraverted personality and tendency toward extraversion.

**Table 2:** Descriptive statistics of patients’ general characteristics.

Variable	Category	Frequency	Percentage
Age (years)	29–30	4	1.8
	31–49	26	12.0
	50–59	69	31.8
	60–74	118	54.4
Sex	Male	197	90.8
	Female	20	9.2
Marital status	Divorced	15	6.9
	Married	202	93.1
Educational level	Junior high school	127	58.5
	Technical secondary school	71	32.7
	University (associate + bachelor’s degree)	19	8.7
Residence	Urban	122	56.2
	Rural	95	43.8
Employment status	Retired	85	39.2
	Unemployed	106	48.8
	Employed	26	12
Monthly family income (CNY)	<3,000	128	59
	3,001–5,000	39	18
	5,001–8,000	42	19.4
	>8,001	8	3.7
Medical payment method	National health insurance	202	93.1
	Self-pay	12	5.5
	Others	3	1.4
Religious belief	None	213	98.2
	Yes	4	1.8

### 3.2 Correlation analysis of extraversion-introversion personality traits, social support, coping style, and self-perceived speech handicap in head and neck cancer patients with tracheostomy

Pearson product–moment correlations were computed among extraversion-introversion (higher scores indicating greater extraversion), positive coping, social support, and SHI score (higher scores indicating greater self-perceived speech handicap). Effect sizes were interpreted following Cohen’s conventions, with |*r*| ≈ 0.10, 0.30, and 0.50 indicating small, medium, and large effects, respectively (Cohen, 1988).

All six correlations were statistically significant (all *p* < 0.01) and fell in the medium range (|*r*| = 0.384–0.481), corresponding to 14.7–23.1% of shared variance. In applied terms: patients with higher extraversion (i.e., less introverted dispositions) tended to report more positive coping (*r* = 0.481, medium-to-large) and greater social support (*r* = 0.386, medium); positive coping and social support were moderately intercorrelated (*r* = 0.384, medium), consistent with the theorized sequential ordering of the two mediators. Higher SHI scores—greater self-perceived speech handicap—were moderately associated with lower extraversion (*r* = −0.445), less positive coping (*r* = −0.410), and lower social support (*r* = −0.402); that is, more introverted, less positively coping, and less supported patients perceived their speech handicap as more severe. These medium-sized associations are large enough to be clinically meaningful, yet not so large as to suggest redundancy among the constructs, supporting their treatment as distinct variables in the subsequent mediation analysis. Detailed statistics are presented in Table 1.

### 3.3 Mediation effect analysis of extraversion-introversion personality traits and self-perceived speech handicap in head and neck cancer patients with tracheostomy

**Figure 1:**
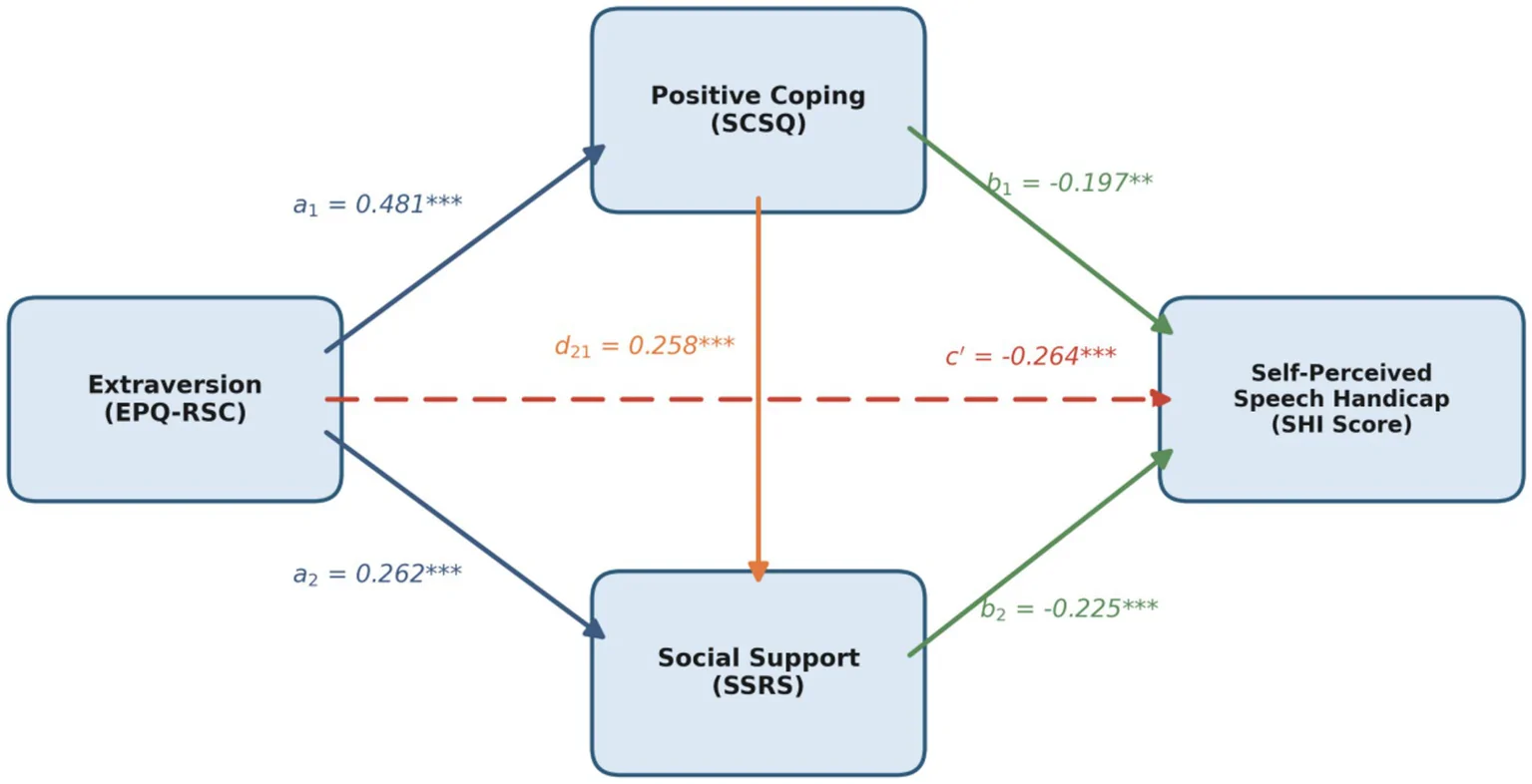
Chain mediation model. Extraversion (EPQ-RSC E scale) → Positive Coping (SCSQ) → Social Support (SSRS) → Self-Perceived Speech Handicap (SHI score). Path coefficients are standardized *β* values adjusted for age, sex, marital status, education, residence, employment status, and family income. The dashed line denotes the direct effect (*c′* = −0.264***); the total effect (*c* = −0.446***) is reported in Table 3. *d*21 denotes the path from positive coping to social support. **p < 0.01, ***p < 0.001. EPQ-RSC = Eysenck Personality Questionnaire-Revised, Short Scale for Chinese; SCSQ = Simplified Coping Style Questionnaire; SSRS = Social Support Rating Scale; SHI = Speech Handicap Index.

A mediation effect model was constructed with coping style and social support as mediating variables, extraversion-introversion personality traits as the independent variable, and self-perceived speech handicap as the dependent variable, as illustrated in Figure 1. We report the magnitude, direction, and proportion of the total effect mediated by each pathway, rather than classifying effects as ‘complete’ or ‘partial’ mediation (Preacher and Hayes, 2008).

Extraversion-introversion personality traits were found to exert a significant negative effect on self-perceived speech handicap (c) in head and neck cancer patients with tracheostomy (*β* = −0.446, *t* = −7.296, *p* < 0.001), indicating that the total effect of extraversion-introversion personality traits on self-perceived speech handicap was significant. Extraversion-introversion personality traits had a significant positive association on the mediating variable coping style (a1) (*β* = 0.481, *t* = 8.052, *p* < 0.001), and a significant positive effect on the mediating variable social support (a2) (*β* = 0.262, *t* = 3.762, *p* < 0.001). Additionally, coping style exerted a significant positive effect on the mediating variable social support (*β* = 0.258, *t* = 3.692, *p* < 0.001), demonstrating that the first half of both mediation pathways was significant. Extraversion-introversion personality traits exhibited a significant negative effect on self-perceived speech handicap (c’) (*β* = −0.264, *t* = −3.874, *p* < 0.001), coping style was negatively associated with self-perceived speech handicap (b1) (*β* = −0.197, *t* = −2.899, *p* < 0.01), and social support also showed a significant negative association with self-perceived speech handicap (b2) (*β* = −0.225, *t* = −3.482, *p* < 0.001). These results indicated that the direct effect of mediation and both latter-half pathways were significant. Collectively, all three mediation effects were confirmed. There is an associative pattern between extraversion–introversion and speech handicap that is potentially explained by the chained mediating roles of coping style and social support (Table 3).

**Table 3:** Testing of the mediation model of coping style and social support between extraversion-introversion personality traits and self-perceived speech handicap.

Variables	Model 1		Model 2		Model 3		Model 4	
	Self-perceived speech handicap (SHI score)		Coping style		Social support		Self-perceived speech handicap (SHI score)	
	*β*	*t*	*β*	*t*	*β*	*t*	*β*	*t*
Extraversion-introversion	−0.446	−7.296***	0.481	8.052***	0.262	3.762***	−0.264	−3.874***
Coping style					0.258	3.692***	−0.197	−2.899**
Social support							−0.225	−3.482***
*R*	0.446		0.481		0.447		0.538	
*R* ^2^	0.198		0.232		0.200		0.289	
*F*	53.224***		64.833***		26.779***		28.842***	

*P < 0.05, **P < 0.01, and ***P < 0.001.

### 3.4 Chain mediation effect analysis of extraversion-introversion personality traits and self-perceived speech handicap in head and neck cancer patients with tracheostomy

**Figure 2:**
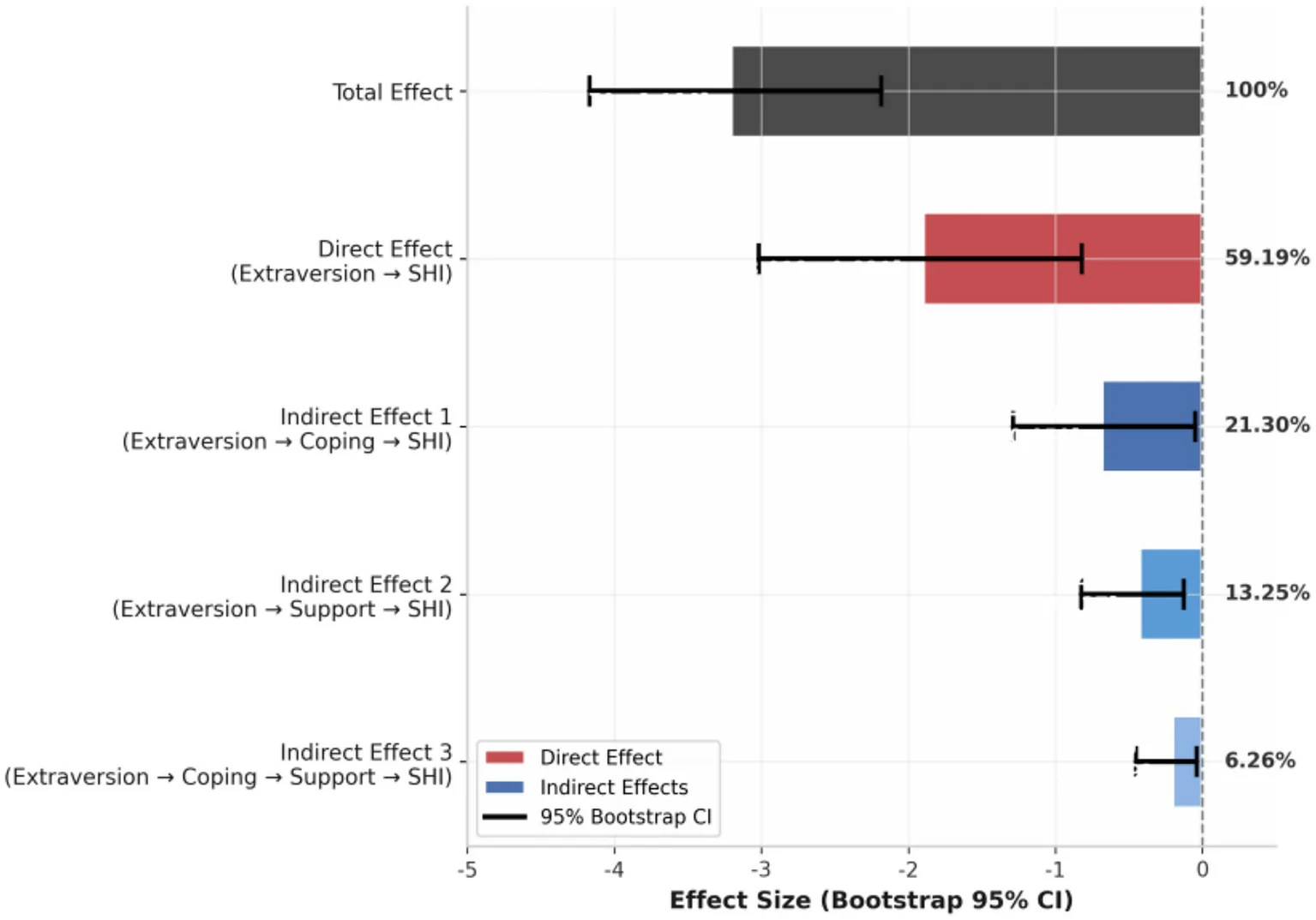
Decomposition of mediation effects. All effects are based on 5,000 bootstrap resamples with bias-corrected 95% confidence intervals. Indirect effect 1 = mediation via positive coping; indirect effect 2 = mediation via social support; indirect effect 3 = chain mediation via positive coping and social support. **p < 0.01 and ***p < 0.001.

The mediation effects of coping style and social support between extraversion-introversion personality traits and self-perceived speech handicap were assessed using the Bootstrap method, with the results provided in Table 4 and Figure 2. The 95% CIs for the upper and lower limits of the three pathways (extraversion-introversion → coping style → self-perceived speech handicap; extraversion-introversion → social support → self-perceived speech handicap; extraversion-introversion → coping style → social support → self-perceived speech handicap) did not include 0. The bias-corrected Bootstrap CI for the “extraversion-introversion → coping style → self-perceived speech handicap” pathway was [−1.288, −0.050]; for the “extraversion-introversion → social support → self-perceived speech handicap” pathway was [−0.825, −0.127]; and for the chain pathway was [−0.451, −0.039]. The bias-corrected CI for direct effects was [−3.018, −0.821], and for total effects was [−4.170, −2.186]. These findings indicate that coping style and social support significantly mediated the association between extraversion-introversion and self-perceived speech handicap.

**Table 4:** Mediation effect test of positive coping style and social support between extraversion-introversion and self-perceived speech handicap.

Effect decomposition		Effect	BootSE	BootLLCI	BootULCI	Effect size proportion
Total effect		−3.204	0.498	−4.170	−2.186	
Direct effect		−1.896	0.551	−3.018	−0.821	59.19%
Indirect effect	TOTAL	−1.307	0.298	−1.889	−0.722	40.81%
	Ind1	−0.682	0.317	−1.288	−0.050	21.30%
	Ind2	−0.425	0.180	−0.825	−0.127	13.25%
	Ind3	−0.201	0.107	−0.451	−0.039	6.26%
	(C1)	−0.258	0.424	−1.030	0.637	
	(C2)	−0.482	0.369	−1.166	0.286	
	(C3)	−0.224	0.165	−0.575	0.081	

BootSE = bootstrap standard error; BootLLCI and BootULCI = lower and upper limits of the bias-corrected 95% bootstrap confidence interval (5,000 resamples). Ind1: Effect size of the mediation pathway “extraversion-introversion personality traits → coping style → self-perceived speech handicap”. Ind2: Effect size of the mediation pathway “extraversion-introversion personality traits → social support → self-perceived speech handicap”. Ind3: Effect size of the mediation pathway “extraversion-introversion personality traits → coping style → social support → self-perceived speech handicap”. (C1): Ind1-Ind2. (C2): Ind1-Ind3. (C3): Ind2-Ind3. C: The difference between two effects, testing whether the difference is significant.

## 4 Discussion

This discussion was organized around the study’s principal findings: sample characteristics, bivariate associations, and the chain-mediation model, followed by alternative explanations and the rationale for the sample composition. Findings from the present data were explicitly marked, and each was followed by its interpretation relative to prior literature.

### 4.1 Current status analysis of extraversion-introversion personality traits and self-perceived speech handicap in head and neck cancer patients with tracheostomy

The results of this study indicated that the most common personality trait within extraversion-introversion dimensions was the intermediate type (44.7%), with T scores approaching the lower threshold of this category, followed by extraverted personality and extraversion-tendency personality, which accounted for 20.28 and 17.51%, respectively. This distribution is consistent with prior evidence that personality shapes the choice of coping strategies in head and neck cancer patients (Aarstad et al., 2012; Aarstad et al., 2007). In this study, male, married, and lower-income participants predominated. In the Chinese sociocultural context, male patients often assume major economic and decision-making responsibilities within households; under combined economic and familial stress, this role burden may intensify patients’ self-perceived burden (Jiang et al., 2019). We note this as sociocultural background rather than a prescriptive claim about gender roles. Health education for head and neck cancer patients with tracheostomy should be reinforced in clinical practice to improve disease-related knowledge, thereby strengthening the modifiable mediators identified in this study—positive coping style and social support—rather than attempting to alter personality traits themselves.

The SHI captures both the perceived severity of speech problems and their psychosocial impact; as a patient-reported measure, it reflects patients’ subjective experience of speech handicap rather than objectively measured speech function (Dwivedi et al., 2011). In this study, the maximum possible SHI score was 120, with a total mean score of (73.230 ± 24.227), indicating an above-average level, which aligns with related research findings (Chen et al., 2024). Patients’ SHI scores indicated marked self-perceived problems with speech function, with etiologies categorized into organic and psychosocial domains, which markedly increased speech handicap severity. Certain surgical procedures result in partial speech dysfunction, and self-perceived speech handicap becomes more pronounced in patients with concurrent tracheostomy, as it is linked to physiological functions such as breathing, speech, mastication, and swallowing involving the oral cavity and trachea (Dawson et al., 2017). Moreover, patients frequently experience varying degrees of physical, behavioral, and emotional fatigue, as well as social isolation, caused by factors such as saliva odor, drooling, and the presence of tracheal tubes. These challenges reduce motivation to engage in social interactions, including communication with healthcare professionals about their condition, thereby further worsening self-perceived speech handicap (Liu et al., 2022). For this reason, the establishment of effective non-verbal communication strategies (e.g., videos, gestures, drawings) prior to surgery is considered essential (Chen et al., 2018). Building on this foundation, individuals with distinct personality traits receive and utilize social support differently, thereby influencing their cognitive appraisal and coping style in relation to disease. Consequently, further investigation into mechanisms affecting postoperative self-perceived speech handicap (e.g., social support and coping style examined in this study) is necessary to enhance communication outcomes in this patient population.

### 4.2 Correlation analysis of postoperative self-perceived speech handicap with extraversion-introversion personality traits, social support, and coping style in head and neck cancer patients with tracheostomy

Before interpreting each association, we note the overall pattern of effect sizes: all correlations were medium in magnitude (|*r*| = 0.384–0.481; 14.7–23.1% shared variance). In psycho-oncology research, where outcomes are multiply determined, medium-sized correlations are typically considered clinically meaningful. This pattern supports two premises of our mediation model: the variables are sufficiently associated to justify testing indirect pathways, yet sufficiently distinct (no pair sharing more than a quarter of variance) to preclude multicollinearity concerns.

This study revealed negative correlations between self-perceived speech handicap and extraversion-introversion personality traits, coping style, and social support. The detailed analysis is as follows: (1) In the present sample, patients with stronger introverted tendencies showed greater self-perceived speech handicap (higher SHI scores; *r* = −0.445). Individuals with introverted personalities are often characterized by pessimism, depression, and irritability in response to stimulation, and some patients also demonstrate loneliness and poor adaptation to the environment (Liu, 2009). Such patients tend to experience more severe postoperative self-perceived speech handicap compared with those possessing extraverted personalities and are more susceptible to depression, consistent with the findings of Xu et al. (2011). (2) In the present sample, patients with lower positive coping showed greater self-perceived speech handicap (*r* = −0.410). Current research on disease self-disclosure among cancer patients with different coping style remains inconclusive. Some scholars argue that negative coping in cancer patients reflects avoidance behavior intended to escape pressures from disease, society, and family (Wang and Hu, 2015). Although such avoidance may increase postoperative speech handicap and hinder communication, it may also serve as a positive coping style mechanism, similar to denial in psychological defense. Opposing views suggest that with the advancement of medical technology and heightened public awareness of health and life, increasing numbers of patients actively seek medical care and cooperate with treatment, implying that surrendering and compromising to disease should not be regarded as normative. Therefore, healthcare professionals are encouraged to adopt more effective strategies to help patients develop improved disease coping style (Zhu et al., 2023b; Cui and Xiao, 2015). This study provides preliminary evidence of the potential utility of addressing coping style and social support in the perioperative period. (3) In this study, patients with lower levels of social support displayed greater postoperative speech handicap, which exerted greater effects on self-perceived speech handicap (*r* = −0.402). Greater material and emotional support derived from social networks enables patients to sustain positive social relationships and engage in more constructive disease coping behaviors (Ma et al., 2020), a finding consistent with the report of Zhu et al. (2023a) on the mediating effect of social support between Traditional Chinese Medicine five-temperament personality and coping style in breast cancer patients. Mishra and Saranath (2019) investigated 143 ovarian cancer patients and demonstrated that when patients discussed their disease with family or friends who responded critically or indifferently, they experienced greater psychological distress and a reduced willingness for disease self-disclosure. Conversely, when supportive and receptive listeners were available, patients were more inclined to engage in self-disclosure.

### 4.3 Chain mediation effect analysis of positive coping style and social support between extraversion-introversion personality traits and self-perceived speech handicap

It is worth noting that the chained mediating effect of social support and positive coping style accounted for only 6.26% of the total effect, indicating a relatively modest proportion compared with the direct effect of extraversion-introversion personality traits (59.19%). However, the existence of this chained mediating effect (rather than its absolute magnitude) verifies the core theoretical hypothesis of this study. By integrating Eysenck’s Revised Personality Theory and Lazarus’ Transactional Model of Stress and Coping, we proposed a chain logic of “personality traits → coping style → social support → self-perceived speech handicap” for head and neck cancer patients with tracheotomy. Existing studies have only explored pairwise correlations between these variables (e.g., personality and social support (Marsh et al., 2019)) but have not integrated them into a unified mediating chain. The significant but modest chained mediating effect (95% bias-corrected bootstrap CI [−0.451, −0.039], excluding zero) indicates that the association between personality traits and self-perceived speech handicap is not entirely direct. In total, 40.81% of the total effect was transmitted indirectly through the two mediators, 21.30% via positive coping and 13.25% via social support, of which 6.26% flowed through the sequential ‘coping → social support’ pathway, i.e., through behavioral strategies (positive coping) that in turn mobilize psychosocial resources (social support). This finding fills the theoretical gap in understanding the “indirect pathway” of personality on self-perceived speech handicap in this specific population, providing empirical support for the applicability of classic personality and stress-coping theories in head and neck cancer nursing. The modest effect size is consistent with the characteristics of psychological and behavioral research in oncology. Personality traits, as stable inherent characteristics, often exert direct and dominant effects on health outcomes, while psychosocial factors (such as social support and coping style) typically play subtle but cumulative regulatory roles. Similar studies in psycho-oncology have reported that chained mediating effects involving psychosocial variables often have relatively small effect sizes, consistent with other chain-mediation studies in cancer patients (Liu et al., 2022; Zhu et al., 2023a). Thus, the 6.26% effect size in this study is within the reasonable range of similar studies.

On the other hand, it was observed that the sample was predominantly male (90.8%) and married (93.1%). Moreover, supportive family systems were found to enhance patients’ confidence in overcoming illness, which is consistent with findings from social support-related studies (Zhu et al., 2023a; Li et al., 2024). Therefore, family caregivers may be actively involved in perioperative support: health education can be provided simultaneously to caregivers, family members, and patients during the perioperative period to reduce self-perceived speech handicap (SHI scores); whether such interventions affect objectively measured speech function remains to be tested. Positive coping style was regarded as a prerequisite for patients to overcome illness. Patients in this study exhibited moderate coping levels, with a mean score of (30.790 ± 7.240) points. In the present sample, more positive coping was associated with lower self-perceived speech handicap; similarly, prior work shows that greater coping positivity is associated with lower illness uncertainty in patients (He et al., 2023). In clinical practice, closer attention should be directed toward patients inclined to surrender to illness, as they are more likely to adopt negative coping style, thereby increasing the risk of greater postoperative speech handicap.

The direct effect accounted for 59.19% of the total effect, whereas the indirect effects via positive coping and social support accounted for 21.3 and 13.25%, respectively, indicating that extraversion-introversion was the principal factor associated with self-perceived speech handicap in these patients. The chained mediation model of this study essentially reflects the theoretical logic of “personality traits → coping strategies → psychosocial adaptation” in head and neck cancer patients with tracheotomy. In this sense, the chain disaggregates the broader construct of psychosocial adaptation into two measurable, sequentially ordered, and intervention-amenable components. Its mechanisms are classic personality theory and stress-and-coping theory, while confirming the particularity of the psychological adaptation process in cancer patients.

As established above, the mediators—but not personality—are intervention-amenable. The clinical implications of this model follow from this asymmetry. Personality therefore serves as a screening marker, and the mediators as intervention entry points. In practice, the Eysenck Personality Questionnaire (EPQ) can be integrated into routine preoperative assessment to develop differentiated support plans. For introverted patients, a “passive support activation” strategy may be adopted, in which nurses proactively connect patients, who are unlikely to seek help themselves, to family companionship and peer-support groups. For extraverted patients, an “active support enhancement” strategy may be implemented, providing diversified support channels such as online rehabilitation communities and communication-skills training. General supportive behaviors, including encouragement and attentive listening, may strengthen patients’ self-confidence and are consistent with nurse-led psychosocial approaches shown to benefit patients with head and neck cancer (van der Meulen et al., 2013). Two further concrete practices map directly onto the two mediators. First, narrative nursing, a structured practice in which nurses elicit and respond to patients’ illness narratives, delivers emotional support while reinforcing positive coping, and thus operates through both mediators. Second, a “nurse–patient–family” collaborative coping model engages family caregivers in patients’ communication rehabilitation (e.g., assisting with alternative communication aids) while providing emotional support, so that family involvement strengthens positive coping through the social-support pathway (van der Meulen et al., 2013). Whether such interventions reduce self-perceived speech handicap should be tested in future interventional studies.

### 4.4 Plausible alternative hypotheses, combining the core variables of our study

Bidirectional relationship between coping and social support: We acknowledge that the cross-sectional design cannot rule out bidirectional associations—on one hand, social support may be associated with the adoption of coping strategies (consistent with our theoretical hypothesis); on the other hand, individuals with more positive coping style may be more likely to actively seek and obtain social support. This bidirectional potential is a key limitation of the current study.

Reverse causality between self-perceived speech handicap and social support: We recognize the plausible alternative hypothesis that self-perceived speech handicap may shape the use of social support (rather than the reverse). For example, individuals with severe communication impairment may face greater barriers in accessing social support, which could further affect their coping strategies. Our current model is based on theoretical prior knowledge, but cross-sectional data cannot verify the direction of this association.

Compliance-based alternative hypothesis (a competing interpretation) also deserves consideration. Introversion is typically linked to avoidance behavior, consistent with the negative association observed between introversion and positive coping in this study. However, introverted patients—being more autonomous and less dependent on social interaction—might show higher compliance with speech rehabilitation and voice-rest advice, whereas extraverted patients’ stronger inclination to communicate might lead them to use their voices prematurely against such advice. Under this alternative account, introversion could confer a behavioral advantage that partially offsets its psychosocial disadvantages. The present cross-sectional data cannot adjudicate between the avoidance and compliance accounts, because they capture the net association between dispositions and perceived handicap at a single time point rather than behavioral trajectories over the recovery course. Future longitudinal studies incorporating objective adherence measures (e.g., therapy attendance, voice-rest compliance) are needed to disentangle these competing mechanisms.

We emphasize that these alternative hypotheses highlight the necessity of future longitudinal studies. Tracking changes in coping, social support, and self-perceived speech handicap over multiple time points (e.g., pre-treatment, post-treatment, 6-month follow-up) can help clarify the temporal sequence of variables and test bidirectional or reverse causal relationships. Additionally, we suggest that future studies could adopt cross-lagged panel models (CLPM) to specifically examine the directional and bidirectional associations between key variables. Cross-lagged panel models estimate, across repeated measurement waves, the extent to which each variable predicts subsequent change in another while controlling for their stability over time, thereby testing the directionality of associations.

### 4.5 Rationale for the skewed sample composition

The study population is specifically head and neck cancer patients with tracheotomy, and the sample composition reflects the clinical reality of this subpopulation in our research setting:

Sex bias (90.8% male): Consistent with epidemiological data, head and neck cancer (including laryngeal, pharyngeal, and oral cavity cancers) has a well-documented male predominance, with a male-to-female incidence ratio of approximately 3:1 to 5:1 globally (Stafford et al., 2016; Ferlay et al., 2021). Moreover, tracheotomy is more frequently performed in patients with advanced-stage head and neck cancer (to address airway obstruction), and male patients are more likely to present with advanced disease due to delayed help-seeking behaviors (Chauhan et al., 2022). Epidemiological studies confirm that this subpopulation has an even higher male predominance than general head and neck cancer patients, due to the aforementioned factors (advanced disease, delayed help-seeking). For example, a large-scale study of 447 head and neck cancer patients reported a male proportion of 89.40% (National Institute for Health and Care Excellence, 2015), consistent with our sample’s 90.8% male ratio. This confirms that our sample’s sex distribution is consistent with the epidemiological characteristics of the target population (head and neck cancer with tracheotomy). Marital status bias (93.1% married): Married patients are more likely to have family support for medical decision-making and long-term care, which increases their willingness to participate in clinical research (Yuan et al., 2020). Unmarried or widowed patients often face greater logistical barriers (e.g., transportation, caregiving) to study participation, leading to underrepresentation. Low socioeconomic status (SES) predominance: This reflects the socioeconomic profile of head and neck cancer patients in our region, where tobacco and alcohol use (key risk factors) are more prevalent among low-SES populations (World Health Organization, 2026).

Potential impact on model robustness: We acknowledge that sex, marital status, and SES are known to moderate illness-related outcomes. Sex differences are mainly attributed to the fact that female patients may adopt more emotion-focused coping strategies, while male patients tend to use problem-focused coping—this may explain why the model’s associations (e.g., between social support and coping style) are more pronounced in our male-dominated sample. SES differences are mainly attributed to the fact that Low-SES patients often have limited access to social support and healthcare resources, which may confound the relationship with coping style (World Health Organization, 2026).

## 5 Conclusion

Among head and neck cancer patients with tracheostomy, extraversion–introversion is significantly associated with self-perceived speech handicap, and this association was partially transmitted through two sequential, modifiable psychosocial pathways: positive coping (21.30% of the total effect) and social support (13.25%), including a significant chained pathway through both mediators (6.26%), while 59.19% of the total effect remained direct. Because personality is relatively stable and not a realistic intervention target, these findings indicate where clinical effort can be most efficiently directed: screening patients for introverted dispositions preoperatively, and then strengthening positive coping and mobilizing social support to reduce self-perceived speech handicap. The chain-mediation model also provides an empirically supported framework linking dispositional antecedents to patient-reported speech handicap in this population. Longitudinal and interventional studies are needed to confirm the temporal ordering of these pathways and to test whether coping- and support-focused interventions causally reduce self-perceived speech handicap.

## 6 Limitation and future directions

Despite the theoretical and practical implications of this study, several limitations should be acknowledged.

First, the cross-sectional design limits causal inference. All variables were collected simultaneously via self-report questionnaires, which only captures the co-occurrence of variables at a single time point. Although our chained mediation model is theoretically grounded in the sequential association of variables, the cross-sectional data cannot definitively rule out reverse causality (e.g., the outcome variable may indirectly associate the antecedent variables) or confounding by unmeasured factors (e.g., disease severity). It is important to note that Harman’s single-factor test, which we used to control for common method bias, addresses measurement bias rather than causal validity—this test ensures that the observed relationships are not driven by response tendencies but does not verify causal links. Future studies should adopt longitudinal cohort designs to track changes in the key variables (e.g., coping tendency and psychological distress) over multiple time points (e.g., pre-treatment, post-treatment, 6-month follow-up) to establish temporal sequences and strengthen causal inference. Alternatively, experimental intervention studies (e.g., targeting positive coping skills training) could be conducted to directly test the causal effects of the proposed mediators.

Second, objective measures of speech intelligibility (e.g., percentage intelligibility assessed by speech therapists, or acoustic parameters) were not collected. Because SHI scores are partly determined by the severity of the underlying speech disorder, the absence of objective intelligibility data represents a potential source of unmeasured confounding, particularly for the pathways involving coping style and social support, since greater disease severity may alter the availability and mobilization of support. We note, however, that personality was modeled as a stable dispositional antecedent that temporally precedes disease onset, which limits the plausibility of confounding of the personality pathway itself; and that the SHI was selected precisely because it captures the perceived, psychosocial dimension of speech problems beyond objective impairment. Future studies should incorporate standardized intelligibility assessments as covariates to disentangle the contributions of objective impairment and perceived handicap. Additionally, the exact postoperative day of questionnaire administration was not recorded. Although all assessments occurred during the in-hospital cannulated period, residual variability in assessment timing within this window may have introduced measurement imprecision.

Third, the sample for this study was obtained from a single center, which may have introduced selection bias. Furthermore, extraversion-introversion represents only one dimension of personality in the Eysenck Personality Questionnaire, and future research is warranted to investigate the effects of other personality traits on self-perceived speech handicap. The sample size of 217 may have limited power to detect small chain mediation effects, particularly for the sequential pathway accounting for 6.26% of the total effect.

Furthermore, the sample was restricted to surgically treated patients with tracheostomy. Patients treated with primary (chemo)radiotherapy, who constitute a substantial proportion of the head and neck cancer population and experience speech impairment through different mechanisms, were not represented. The findings should therefore not be generalized to non-surgical populations, and future studies should examine whether the proposed pathways operate similarly in patients managed with (chemo)radiotherapy.

Although the mediation model adjusted for age, sex, marital status, education, residence, employment status, and household income, other clinically relevant covariates—particularly disease stage, and surgical extent—were not available for adjustment and may confound the observed associations. Future studies should incorporate these covariates and ensure adequate statistical power.

The current study did not directly measure anxiety, depression, or resilience—variables that may further mediate/moderate the proposed relationships. For example, extraversion might be associated with lower handicap through resilience, or anxiety might moderate the coping–handicap association—possibilities suggested by the wider stress-and-coping literature (Connor-Smith and Flachsbart, 2007; Carver and Connor-Smith, 2010) but untestable in the present dataset.

Despite these limitations, the current study provides preliminary evidence for the chained mediation model, highlighting the sequential pathways linking antecedent variable to outcome variable via mediator. These findings offer a theoretical basis for developing targeted psychological interventions for head and neck cancer patients (e.g., enhancing positive coping to reduce negative emotions and improve treatment adherence). Future research addressing the above limitations will further validate the causal relationships and strengthen the practical application of the findings.

## Data Availability

The original contributions presented in the study are included in the article/supplementary material, further inquiries can be directed to the corresponding author.
